# Naturally Occurring Differences in CENH3 Affect Chromosome Segregation in Zygotic Mitosis of Hybrids

**DOI:** 10.1371/journal.pgen.1004970

**Published:** 2015-01-26

**Authors:** Shamoni Maheshwari, Ek Han Tan, Allan West, F. Chris H. Franklin, Luca Comai, Simon W. L. Chan

**Affiliations:** 1 Department of Plant Biology and Genome Center, University of California, Davis, Davis, California, United States of America; 2 School of Biosciences, University of Birmingham, Edgbaston, Birmingham, United Kingdom; 3 Department of Plant Biology, University of California, Davis, Davis, California, United States of America; 4 Howard-Hughes Medical Institute and the Gordon and Betty Moore Foundation, University of California, Davis, Davis, California, United States of America; Harvard University, UNITED STATES

## Abstract

The point of attachment of spindle microtubules to metaphase chromosomes is known as the centromere. Plant and animal centromeres are epigenetically specified by a centromere-specific variant of Histone H3, CENH3 (a.k.a. CENP-A). Unlike canonical histones that are invariant, CENH3 proteins are accumulating substitutions at an accelerated rate. This diversification of CENH3 is a conundrum since its role as the key determinant of centromere identity remains a constant across species. Here, we ask whether naturally occurring divergence in CENH3 has functional consequences. We performed functional complementation assays on *cenh3-1*, a null mutation in *Arabidopsis thaliana*, using untagged CENH3s from increasingly distant relatives. Contrary to previous results using GFP-tagged CENH3, we find that the essential functions of CENH3 are conserved across a broad evolutionary landscape. CENH3 from a species as distant as the monocot *Zea mays* can functionally replace *A. thaliana* CENH3. Plants expressing variant CENH3s that are fertile when selfed show dramatic segregation errors when crossed to a wild-type individual. The progeny of this cross include hybrid diploids, aneuploids with novel genetic rearrangements and haploids that inherit only the genome of the wild-type parent. Importantly, it is always chromosomes from the plant expressing the divergent CENH3 that missegregate. Using chimeras, we show that it is divergence in the fast-evolving N-terminal tail of CENH3 that is causing segregation errors and genome elimination. Furthermore, we analyzed N-terminal tail sequences from plant CENH3s and discovered a modular pattern of sequence conservation. From this we hypothesize that while the essential functions of CENH3 are largely conserved, the N-terminal tail is evolving to adapt to lineage-specific centromeric constraints. Our results demonstrate that this lineage-specific evolution of CENH3 causes inviability and sterility of progeny in crosses, at the same time producing karyotypic variation. Thus, CENH3 evolution can contribute to postzygotic reproductive barriers.

## Author Summary

As populations evolve into new species they acquire mutations that are compatible with their own genetic background, but often lead to defects when crossed to others. Here, we show that naturally evolved differences in the centromere-specific histone H3 (CENH3) can contribute to this process. Unlike canonical histones, CENH3 differentiates rapidly even between closely related species. To better understand the functional role of natural CENH3 variation, we complemented a null allele of Arabidopsis with progressively more distant orthologs. Contrary to previous findings, we discovered that all tested variants, even the highly diverged maize CENH3, could restore normal growth and reproduction in selfing individuals. However, when crossed to the wild type, hybrid progeny suffered from extensive mis-segregation. Genotypes include simple aneuploids, novel genetic rearrangements, and in extreme cases haploids where all the chromosomes from one parent are lost. This indicates that while wide variation in CENH3 is compatible with its essential function, epigenetically different centromeres do not function well when brought together in a hybrid embryo. A better understanding of haploid generation would have profound effects on plant breeding and our results suggest that the natural variation of CENH3 could offer a cache of testable variation.

## Introduction

Centromeres are the site where spindle microtubules attach to chromosomes during cell division. This attachment is mediated via a multi-protein complex called the kinetochore, a structure essential for the stable inheritance of genetic information. Contrary to expectation, the centromere is not a genetic locus in the traditional sense of being defined by its DNA sequence [1,2]. The DNA sequence underlying the centromere is not evolutionarily conserved and in most species, is composed of megabases of rapidly evolving tandem repeats [3]. However, these repeats are not essential to centromere formation since neocentromeres or the gain of new centromeric activity has been observed over unique DNA sequences as well [4–6]. The common denominator to all centromeres, old and new, is the presence of a centromere specific histone variant of H3 called CENH3 (or CENP-A) [7]. This and other evidence [8–10] indicate that in both plants and animals, the location of centromeres is specified epigenetically by the presence of CENH3.

Despite this ancient and conserved role of CENH3 in maintaining genetic integrity, the CENH3 protein sequence is not evolving under purifying selection. In contrast to the nearly invariant histone H3, CENH3 homologs are highly divergent. For example, CENH3 from *Arabidopsis thaliana* and *Arabidopsis arenosa*, sister species that shared a common ancestor approximately 5 MYA, differ at 23 of 178 amino acid positions while canonical Histone H3 has accumulated only 4 substitutions out of 136 amino acid positions since the divergence of plants and animals. In the *Brassicaceae* and in *Drosophila*, the diversification of CENH3 at both the Histone Fold Domain (HFD) and the N-terminal tail appears to be driven by adaptive evolution under natural selection [11,12]. This accelerated evolution is especially pronounced at the N-terminal tail of CENH3, which is hyper-variable both in its length and sequence. Why a structure essential for stable inheritance of genetic material is composed of genetically unstable units is a fundamental unsolved question in the field of chromosome biology.

The “centromere drive” hypothesis proposed by Henikoff and Malik puts forward genetic conflict as the source of this striking diversification [13]. This model supposes that DNA sequence can influence centromere function. Female meiosis in animals and plants is asymmetric, in that only one product survives to become the egg cell. If a sequence variant evolves that can preferentially segregate into the surviving egg cell, it will rapidly sweep through the population [14,15]. However, such driving chromosomes would be associated with fitness costs including fixation of linked deleterious mutations, sterility due to non-disjunction and in the case of sex chromosomes, skewed sex ratios. This in turn is expected to set off the evolution of centromere-associated proteins to suppress the selfish transmission of this centromere. Cycles of centromere drive and suppression could result in the rapid diversification of centromeres and associated factors. One outcome of divergence in centromere components, DNA and/or proteins, could be the evolution of incompatibilities in the segregation machinery, leading to the reproductive isolation of populations.

While there is strong evidence attributing expansion of centromeric repeats to meiotic drive [16], whether CENH3 or other centromeric proteins are co-evolving with DNA sequences to suppress instances of drive remains speculative. The functional consequences of CENH3 divergence are difficult to address because CENH3 is an essential gene and most model systems cannot tolerate the segregation errors caused by mutations or modification to its function. In *D*. *melanogaster* and mammalian cells, RNAi has been used to down-regulate CENH3 levels [17,18]. However, the interpretation of any loss-of-function phenotypes is confounded by the persistence of CENH3 through multiple rounds of cell division. In contrast, a *cenh3* null mutant in *A*. *thaliana* allows us to completely replace the endogenous protein with transgenic variants. In addition, *A*. *thaliana* has high-copy centromeric repeats similar in organization to most plants and animals [19], making it an attractive system for testing general principles of centromere function.

Also unique to *A*. *thaliana* is the CENH3-mediated genome elimination system [20], which we have leveraged as a sensitive genetic assay for centromere function in this study. This genetic assay is based on the discovery that when a *cenh3* null mutant expressing a GFP-tagged chimeric CENH3 (GFP-tailswap) is crossed to a wild type, missegregation of chromosomes from the GFP-tailswap parent is observed [20]. Since *A*. *thaliana* has a high tolerance to aneuploidy, the F1 progeny capture a wide range of segregation errors. In the most extreme cases, all the chromosomes from the GFP-tailswap parent are lost (genome elimination) yielding haploid offspring that inherit chromosomes only from the wild-type parent. Importantly, segregation errors are only observed in crosses to wild type and not during normal vegetative growth or when GFP-tailswap plants are selfed. This implies that chromosome missegregation in the F1 zygote is the result of competition between wild-type centromeres and defective centromeres built on the artificial chimeric CENH3. Thus, the frequency of segregation errors and genome elimination can be used as a sensitive assay for centromere function. We were interested in asking what would happen if instead of using an artificial chimeric construct we simply replaced the endogenous CENH3 with natural variants from related species.

Previous studies using GFP-tagged versions of CENH3 orthologs had found a very narrow evolutionary window of functional complementation [21]. This leads to the conclusion that plant CENH3s are evolving under unique and highly dissimilar lineage-specific functional constraints [21]. Here, using untagged natural variants of CENH3 we observed the following: 1) Despite extensive sequence divergence, the essential functions of CENH3 are conserved across a much broader evolutionary time-scale than previously thought; 2) Naturally evolved divergence in CENH3 can contribute to genetic instability by causing chromosome missegregation, generating not only aneuploids and haploids, but also novel genetic rearrangements; 3) It is the divergence in the fast evolving N-terminal tail domain that is responsible for segregation defects and 4) The N-terminal tail appears to be evolving in a modular fashion. With these results, we argue that the core functions of CENH3 have remained unchanged over long evolutionary periods while the N-terminal tail of CENH3 is evolving as a species-specific optimized platform for centromere organization. Finally, our study presents the first direct evidence for the role of CENH3 divergence in speciation.

## Results

### Mustard family *CENH3s* complement Arabidopsis *cenh3–1* null mutation

*A*. *thaliana* is a member of the mustard family (*Brassicaceae*), known for its agriculturally important Brassica crops. Analysis of CENH3 homologs from several species within the mustard family revealed that it is adaptively evolving, both at the Histone Fold Domain (HFD) and the N-terminal tail (NTT) [11]. Ravi *et al*. (2010) [21] had assayed CENH3s from species within the *Brassicaceae* and beyond for functional complementation of *cenh3–1*, a CENH3 null mutation in *A*. *thaliana*. They found that GFP-tagged CENH3 from *Brassica rapa* and *Zea mays* localized at *A*. *thaliana* centromeres, but only GFP-tagged CENH3 from the closely related species *A*. *arenosa* rescued embryo lethality of the *cenh3–1*. A caveat to these experiments was the presence of the GFP-tag. GFP-tagged *A*. *thaliana* CENH3 largely complemented the functions of the *A*. *thaliana cenh3–1* mutation, but when crossed to wild type segregation errors were observed at a low frequency. This hinted that the GFP-tag is not entirely neutral. Thus, to assay only the effects of naturally evolved variation on CENH3 function, we decided to test complementation of the *cenh3* null mutant using native untagged proteins.

We chose CENH3 from *B*. *rapa* and *Lepidium oleraceum*, two species nested within the *Brassicaceae* family. *L*. *oleraceum* is more closely related to *A*. *thaliana* than *B*. *rapa*, but more distantly than *A*. *arenosa* [22]. To test for complementation, we transformed *cenh3–1/CENH3* heterozygotes with constructs expressing genomic sequence encoding *L*. *oleraceum* CENH3 (LoCENH3) and *B*. *rapa* CENH3 (BrCENH3) under the endogenous *A*. *thaliana* CENH3 promoter. We recovered transformants that were homozygous for the *cenh3–1* mutation for both variants in the T1 generation. This result is revealing in two ways: firstly it shows that the GFP-tag interferes with CENH3 function and secondly, it indicates that the previously defined boundary of functional complementation is incorrect.

**Figure 1:**
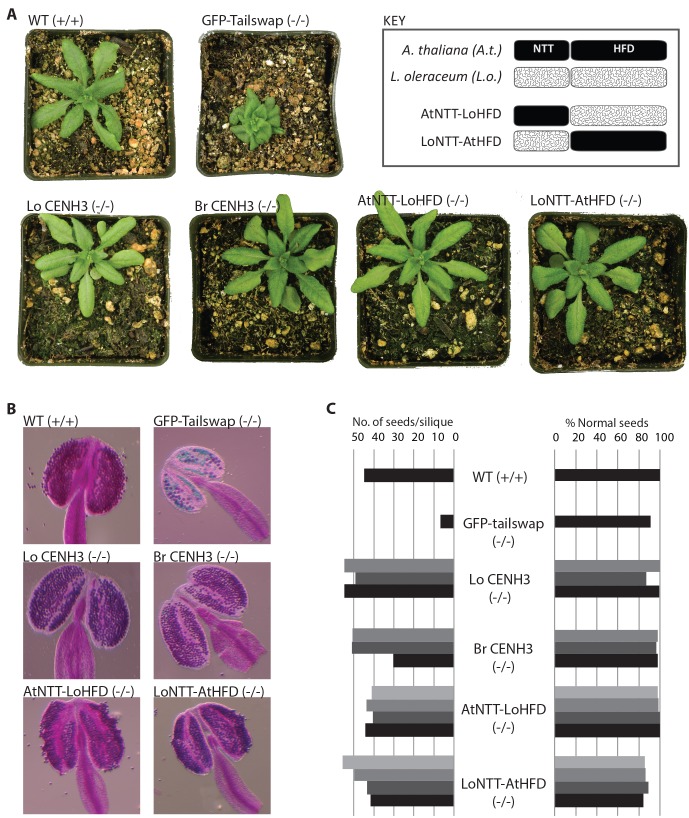
Vegetative and reproductive phenotypes of CENH3 complemented lines. (A) Plants at rosette stage from different complemented lines compared to wild-type Columbia (WT) and GFP-tailswap, a high frequency haploid inducer [20]. The genotype of the endogenous *CENH3* locus is indicated in parentheses. LoCENH3 is *L*. *oleraceum* CENH3 and BrCENH3 is *B*. *rapa* CENH3. AtNTT-LoHFD and LoNTT-AtHFD are chimeric CENH3s described in the key. (B) Anthers stained for viability with Alexander stain. Viable pollen granules stain purple. (C) Measures of fertility based on number of seeds per silique and seed appearance. Bars in different shades of grey represent counts from different T1 lines. For each measurement, seeds from 5 siliques were pooled and counted.

We further characterized the extent of mitotic and meiotic complementation in the T2 generation. *A*. *thaliana* plants homozygous for *cenh3* null mutation expressing transgenic *L*. *oleraceum* CENH3 or *B*. *rapa* CENH3 were phenotypically indistinguishable from wild type (Fig. 1A). We therefore conclude that *B*. *rapa* and *L*. *oleraceum* CENH3 can fully complement *A*. *thaliana* CENH3 mitotic functions required for vegetative growth. Transgenic lines for both CENH3 variants in a *cenh3–1* homozygous background were also self-fertile.

To assay meiotic complementation, we wanted to identify plants that were homozygous for both the *cenh3* null mutation and variant CENH3 transgene. Following segregation ratios of the transgene is not informative in a *cenh3–1* homozygous mutant background, since individuals without transgenic CENH3 cannot survive. Therefore, we decided to use frequency of seed death in selfed siliques of T2 plants to infer the zygosity of the CENH3 transgene. Individuals that are *cenh3* -/- and heterozygous for the transgene are expected to produce 25% seed death upon selfing. Assuming that the transgene is inserted at a single locus, individuals homozygous for the transgene are expected to produce 0 to less than 25% seed death if fully or partially complementing the meiotic functions of the endogenous *A*. *thaliana* CENH3.

Using this criterion to infer the zygosity of the transgene, we measured fertility of *A*. *thaliana* plants in which the endogenous CENH3 is replaced by *L*. *oleraceum* CENH3 or *B*. *rapa* CENH3. We measured seed set and frequency of abnormal seeds in selfed siliques from three independent transformation events for each construct (Fig. 1C). The complemented lines were comparable to wild type for both measures of fertility. Furthermore, viability-stained anthers from the same complemented lines showed live pollen numbers and appearance indistinguishable from wild type (Fig. 1B).

**Figure 2:**
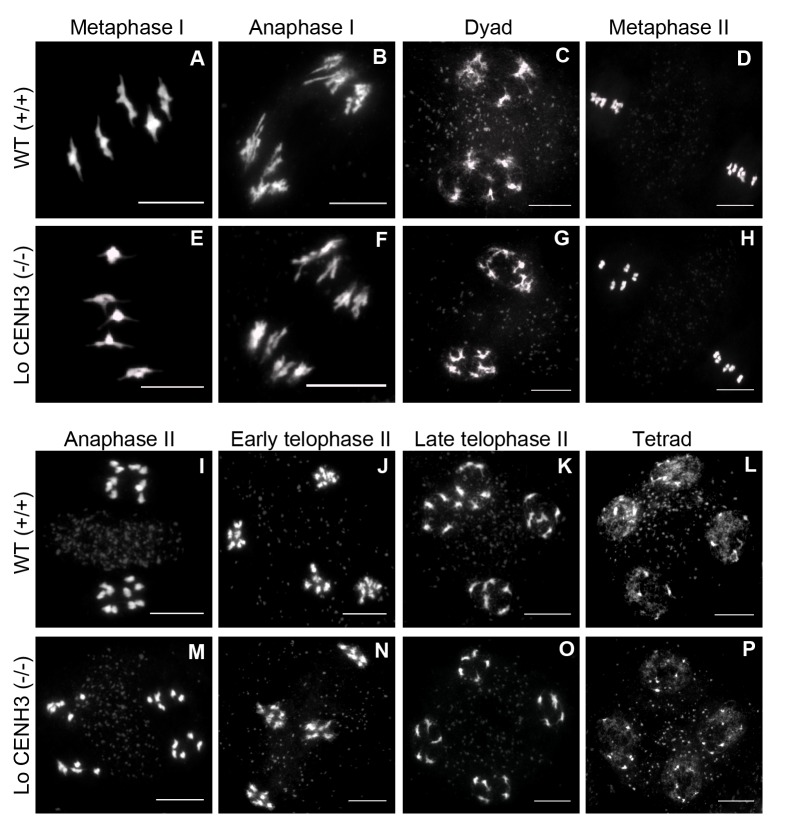
*L*. *oleraceum* CENH3 complements meiosis in *A*. *thaliana*. Male meiotic chromosome spreads stained with DAPI for WT Col-0 (A-D, I-L) and *L*. *oleraceum* CENH3 *cenh3–1/cenh3–1* (T1 family = 19) (E-H, M-P). Scale bar = 10μm.

For *L*. *oleraceum* CENH3 complemented lines we further analysed meiosis cytologically with DAPI stained chromosome spreads from pollen mother cells (PMCs) in two T1 families, 2 and 19. Prophase I of meiosis in both lines was indistinguishable from wild type (S1A Fig.). Chromosome segregation in PMCs at both meiotic divisions was checked for segregation errors. In the T1 = 19 family, metaphase I (n = 26), anaphase I (n = 7), metaphase II (n = 40), anaphase II (n = 5) and telophase II (n = 21) PMCs were scored, none of which displayed segregation errors (Fig. 2). Careful inspection of all post-prophase I PMCs sampled revealed some limited chromosome fragmentation in one anaphase II cell (S1B Fig.). Although, the origin of this cannot be ascertained at present, its low frequency is unlikely to compromise fertility. Thus, we conclude that CENH3 orthologs can complement the essential mitotic and meiotic functions of *A*. *thaliana* CENH3 under standard growing conditions.

### Naturally evolved divergence in CENH3 can cause genome elimination

Next, we wanted to test how *A*. *thaliana* centromeres built on CENH3 variants functioned in comparison to those built on the native *A*. *thaliana* CENH3. To do so, we crossed them as females with pollen from wild-type (CENH3 +/+) Landsberg *erecta* (L*er*) homozygous for the *gl1–1* glabrous mutation, which confers a trichomeless phenotype. We chose L*er* as the CENH3 wild-type parent because the complemented lines were generated in the Col-0 accession. This allows us to use polymorphisms between Col-0 and L*er* to determine the parent of origin for all the chromosomes in the F1. In a standard cross we expect only F1 diploid hybrids with trichomes. However, if replacing the endogenous CENH3 with natural variants creates weak centromeres, then we can expect mitotic missegregation in the F1 zygote.

The first indication of abnormal segregation in these crosses was the observation that 14–47% seeds aborted during development (Table 1). In contrast to the uniformly tan-colored plump seeds generated when the complemented lines are selfed, dark nearly black shriveled seeds were seen in crosses to wild type. Upon germination of F1 seeds from *L*. *oleraceum* CENH3 and *B*. *rapa* CENH3 crosses, we recovered diploid, aneuploid and haploid progeny. All haploids were sterile and paternal on the basis of having a trichomeless appearance, an expression of the recessive *gl1–1* mutation. We confirmed the haploid genome content of 11 phenotypically selected haploids by flow cytometry (S2 Fig.).

**Table 1:** Natural variation in CENH3, specifically in the N-terminal tail, causes genome elimination.

Transgene	T1 family name	% normal seed	Total No. of Plants Analysed	Haploids (%)	Diploids (%)	Aneuploids (%)
GFP-tailswap	11	20 (n = 1187)	606	240 (40)	167 (28)	199 (32)
*L*. *oleraceum* CENH3	2	58 (n = 464)	552	18 (3)	480 (87)	54 (10)
	19	53 (n = 167)	133	15 (11)	93 (70)	25 (19)
	21	86 (n = 294)	529	10 (2)	490 (93)	29 (5)
*B*. *rapa* CENH3	1	70 (n = 180)	283	5 (2)	243 (86)	35 (12)
	3	65 (n = 200)	246	2 (1)	219 (89)	25 (10)
	9	84 (n = 304)	464	4 (1)	445 (96)	15 (3)
AtNTT-LoHFD	4	83 (n = 138)	38	0 (0)	35 (92)	3 (8)
	8	97 (n = 393)	249	0 (0)	230 (92)	19 (8)
	9	92 (n = 364)	117	0 (0)	113 (97)	4 (3)
	24	97 (n = 385)	150	0 (0)	150 (100)	0 (0)
LoNTT-AtHFD	1	10 (n = 403)	119	2 (2)	94 (79)	23 (19)
	3	0 (n = 236)	0	N. A.	N. A.	N. A.
	6	1 (n = 152)	5	0 (0)	4 (80)	1 (20)
	19	2 (n = 334)	0	N. A.	N. A.	N. A.

Crosses were between *cenh3–1/cenh3–1* + CENH3 transgene females and pollen from wild type Landsberg CENH3 +/+ strain homozygous for the *gl1–1* glabrous mutation. Sterile offspring expressing the recessive *gl1–1* trichomeless phenotype were scored as paternal haploid. Offspring with developmental defects were scored as aneuploid. Fertile wild-type offspring were scored as diploid.

For each CENH3 construct we tested two individuals from each of the three independent transformation events (T1 families) in crosses to wild-type L*er gl1–1*. Substantial variation in the frequency of haploids was observed between the different T1 families (Table 1). While the source of this variability is unclear, it is consistent with the variable haploid induction rates observed when GFP-tailswap is crossed to wild type. In cases where *cenh3–1* is complemented with *L*. *oleraceum* CENH3, the frequency of genome elimination ranged from 2 to 11%. For *B*. *rapa* CENH3 complemented *cenh3–1*, the range was 1 to 2%. Although, *L*. *oleraceum* is more closely related to *A*. *thaliana* than *B*. *rapa*, substituting endogenous *A*. *thaliana* CENH3 with the *L*. *oleraceum* ortholog in an *A*. *thaliana* plant appeared to have a greater destabilizing effect on *A*. *thaliana* centromere as inferred from the larger frequency of genome elimination on average (6 ± 2.4% vs. 1 ± 0.2%).

We have not observed any instances of aneuploidy and haploidy in the selfed progeny of the complemented lines (S3 Fig.). In addition meiosis in *L*. *oleraceum* T1 families 2 and 19, which generated the highest frequency of haploids and aneuploids, is wild type in appearance (Fig. 2). From the absence of meiotic defects during selfing, we infer that the segregation errors and genome elimination observed in the crosses to wild type (CENH3 +/+) are not the byproduct of meiotic dysfunction in the inducer parent, but rather the consequences of postzygotic interactions in the hybrid embryo. From this we conclude that natural variation in CENH3 can cause centromere-mediated genome elimination and contribute to genetic instability through changes in ploidy.

### Crosses between plants expressing CENH3 variants and the wild type generate novel genetic rearrangements

**Figure 3:**
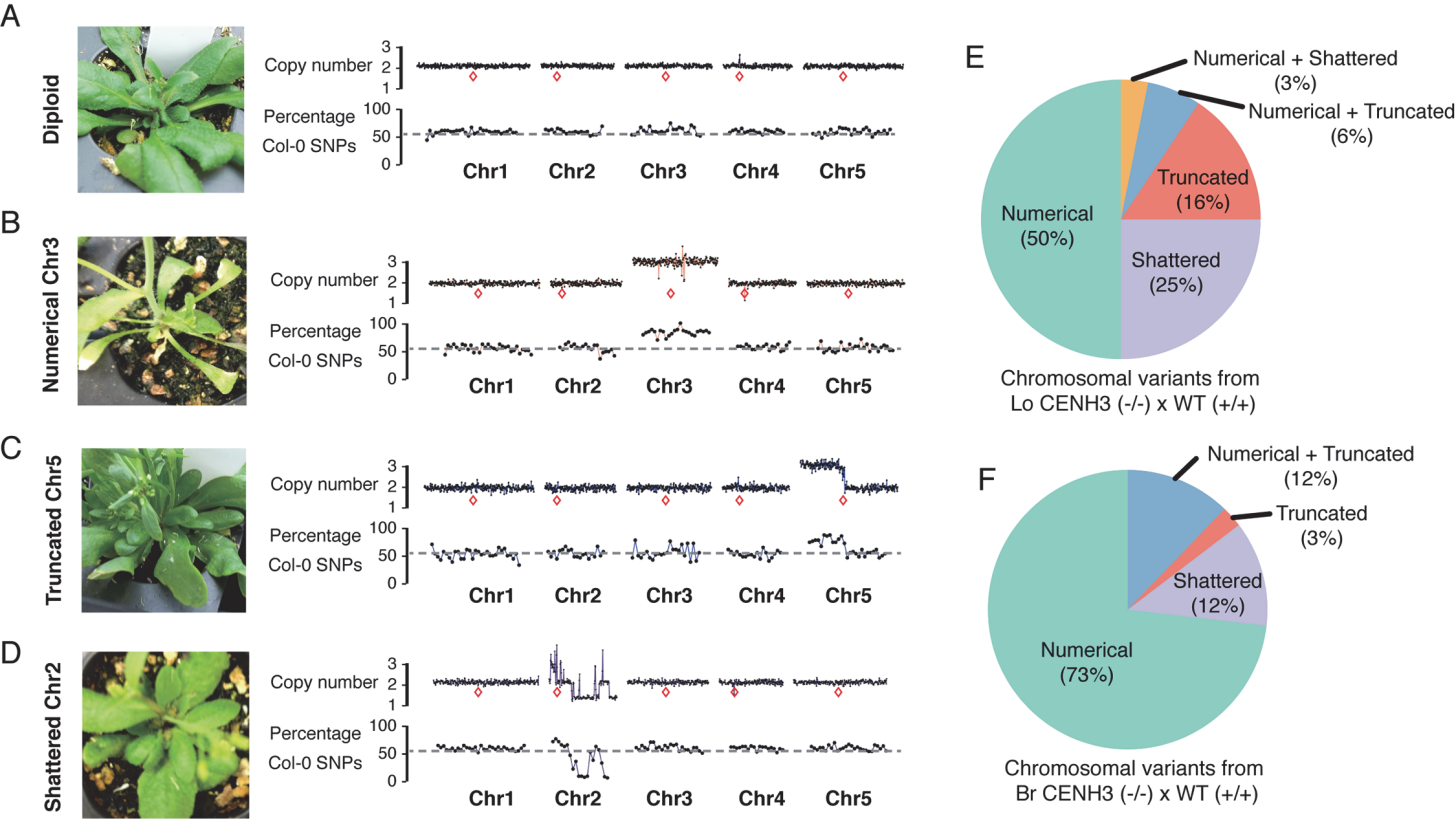
Characterization of aneuploid genotypes using whole-genome sequencing. Shown here are pictures of an individual plant alongside its 100kb bin dosage plot and 1 Mb bin SNP analysis across all five chromosomes. The red boxes indicate their relative centromere positions. (A) A diploid Col-0/L*er* hybrid individual from a genome elimination cross mediated by *Lo*CENH3. (B–D) The three major aneuploid types represented by examples of each: an individual with a numerical aneuploid chromosome (B), a truncated aneuploid chromosome (C) and a shattered aneuploid chromosome (D). (E–F) Percentage of each type of chromosomal variants of the aneuploids derived from a LoCENH3 (E) and BrCENH3 (F) genome elimination cross.

One of the hallmarks of centromere-mediated genome elimination is the generation of aneuploid progeny at a relatively high frequency (~30% for GFP-tailswap) [20]. Aneuploids have imbalanced karyotypes that perturb gene dosage, with large and variable phenotypic consequences. *A*. *thaliana* aneuploids exhibit morphological phenotypes in a wide variety of traits including abnormal leaf morphology, irregular branching patterns and infertility [23]. Using these criteria, we estimated that in crosses of L*er gl1–1* (as the wild-type pollen parent) to *L*. *oleraceum* CENH3 and *B*. *rapa* CENH3 complemented lines, the incidence of aneuploidy is 11.3% and 8.3% respectively (Table 1). We selected 48 phenotypically aneuploid progeny from each cross for whole genome sequencing to determine the relative dosage of each chromosome using a bioinformatics approach. Chromosomes and subchromosomal regions that vary from the expected number of 2 can be readily identified by increased or decreased read count relative to the rest of the genome [23]. We identified chromosomal imbalances in 73 of the 96 individuals selected for sequencing (S4 Fig., S5 Fig. and S1 Table). In this dataset we found three classes of aneuploid chromosome types and an example of each is shown in Fig. 3 (B–D). As a comparison diploid Col/L*er* individual with 2 copies of each of the five *A*. *thaliana* chromosome is shown in Fig. 3A. The first class contains numerical aneuploids where whole chromosomes are duplicated, as exemplified by an individual trisomic for Chr3 (Fig. 3B). The second class contains aneuploids with truncated chromosomes, such as, for example, an extra copy of Chr5 with a truncated left arm (Fig. 3C). Lastly, the third class displays dosage variation consistent with chromosomes that shattered and have gained or lost DNA segments multiple times across the entire length of the chromosome. An example for a shattered Chr2 is shown in Fig. 3D. Based on our low pass sequencing analysis we cannot infer the chromosomal organization of these dosage variants presented here.

Using SNPs between the parental lines, we were able to infer the origins of the copy variant regions (SNP plots in Fig. 3A–D). In all three classes of dosage variants, the DNA contributing to the increased copy number originated from the transgenic Col-0 parent, in which the endogenous CENH3 had been replaced by an evolutionary variant. We even observed the loss of heterozygosity in the shattered Chr2 (Fig. 3D), as a result of the complete loss of the Col-0 chromosomal regions. The largest fractions of aneuploids from these crosses were products of whole chromosome missegregation events (Fig. 3E and F). However, there were also a considerable number of aneuploids with sub-chromosomal changes in copy number. This variation in dosage implies the creation of novel genetic karyotypes.

In summary, centromeres built on CENH3 variants appear to missegregate in crosses to wild type. One consequence of which is aneuploidy and segmental dosage variants and with that the introduction of a broad range of phenotypic diversity [24].

### Essential functions of CENH3 are conserved between monocots and dicots

**Figure 4:**
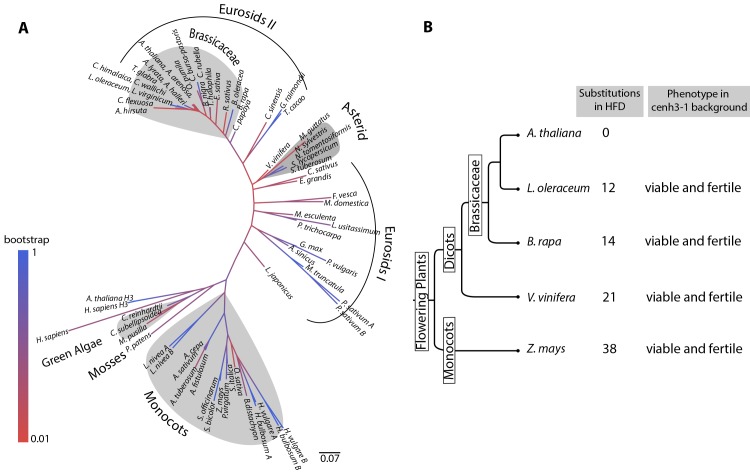
Analysis of evolutionary divergence in plant CENH3 Histone Fold Domains. (A) Phylogenetic tree inferred by using the Maximum Likelihood method based on the JTT matrix-based model [52]. The tree with the highest log likelihood (-3935.2849) is shown. The tree is drawn to scale, with branch lengths measured in the number of substitutions per site. (B) Summary of complementation tests of *A*. *thaliana cenh3–1* mutation with CENH3 from increasingly distant plant species.

Since our results negated the previously identified limits of CENH3 functional complementation, we decided to sample a larger evolutionary space. Flowering plants are divided into two major groups: monocots and dicots that diverged from each other 146–161 MYA. Rosids are the largest clade within the dicots, comprising of around 70,000 species including the model plant *A*. *thaliana* [25]. To better understand the extent of variation in CENH3 across the plant kingdom, we collated 67 CENH3 sequences from public databases that included homologs from green algae, mosses, monocots and dicots (S2 Table). Using protein sequence from the HFD we generated a multiple sequence alignment and constructed a phylogeny of CENH3 in the plant kingdom (Fig. 4A). This CENH3-HFD based gene tree was largely congruent with the accepted evolutionary relationships between these species (Fig. 4A). The most striking feature of the tree is the size of its branches and the variation in their lengths, illustrating the rapid and variable rates of CENH3 evolution. We chose to test CENH3 from two additional species at increasing degrees of evolutionary distance from *A*. *thaliana*: grapevine (*Vitis vinifera*), one of the earliest diverging rosid species considered a basal rosid, and corn (*Zea mays*), a monocot.

To test the functional complementation of these distant species, we made constructs expressing *V*. *vinifera* CENH3 and *Z*. *mays* CENH3 cDNA under control of the endogenous *A*. *thaliana* CENH3 promoter. These transgenes were transformed into *cenh3–1*/CENH3 heterozygotes. We recovered both *V*. *vinifera* CENH3 and *Z*. *mays* CENH3 transformants in a *cenh3–1* homozygous background in the T1 generation (Fig. 4B and S6 Fig.). *V*. *vinifera* and *Z*. *mays* CENH3 have 21 and 38 amino acid substitutions respectively, relative to the 97 amino acid positions in the HFD of *A*. *thaliana* CENH3 (S7 Fig.). Hence, it was surprising that both *V*. *vinifera* CENH3 and *Z*. *mays* CENH3 were able to complement the embryo lethality of the *cenh3–1* resulting in plants undistinguishable from the wild type. To the extent that the complemented lines were self-fertile, we can say that both variants also fulfilled the essential meiotic functions of *A*. *thaliana* CENH3 (Fig. 4B and S6 Fig.).

### Divergence in the N-terminal tail of CENH3 causes missegregation

The *L*. *oleraceum* CENH3 gene has 12 amino acid substitutions in its HFD relative to *A*. *thaliana* and 31 in its N-terminal tail. We generated chimeric proteins in which the N-terminal tail of *L*. *oleraceum* CENH3 was fused to the HFD of *A*. *thaliana* CENH3, and *vice versa* (Fig. 1A). We assayed complementation of *cenh3–1* and found that both chimeras complemented the embryo lethality of the *cenh3–1* mutation in the T1 generation. The chimeric CENH3s were also similar to wild type with respect to pollen viability as determined by viability staining and in number and appearance of developing seeds within siliques (Fig. 1B and 1C).

We then tested the functionality of centromeres built on these chimeric CENH3 transgenes by making crosses to wild type. It was immediately apparent by visual inspection of the resulting F1 seeds that the two chimeras had entirely different effects. The F1 seeds from the chimera with *A*. *thaliana* N-terminal tail fused to *L*. *oleraceum* HFD (AtNTT-LoHFD) crossed to wild type appeared largely normal while most of the F1 seeds from the *L*. *oleraceum* N-terminal tail fused to *A*. *thaliana* HFD (LoNTT-AtHFD) were abnormal in appearance (Table 1). We failed to obtain F1 seed germination from crosses of LoNTT-AtHFD to the wild type except from a single T1 family. In this respect, the function of the chimera, LoNTT-AtHFD, is reduced compared to the full-length *L*. *oleraceum* CENH3. We only recovered 124 F1 progeny from the LoNTT-AtHFD cross, of which 2 were haploids and 23 were phenotypically aneuploid. In contrast, we recovered a large number of F1 progeny from the crosses with AtNTT-LoHFD. However, out of a total of 554 F1’s none were haploids. This indicates that restoring the N-terminal tail to the endogenous sequence is sufficient to restore activity to a level similar to wild-type.

### Evidence for modular evolution of the N-terminal tail within the plant kingdom

**Figure 5:**
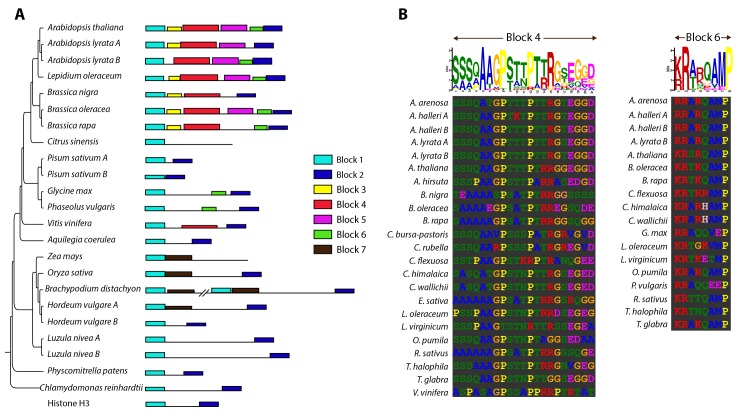
Identification of sequence motifs in plant CENH3 N-terminal tails. (A) Schematic representation of CENH3 N-terminal tails from a subset of plant species, in the context of their known phylogenetic relationships. Motifs identified by MEME [51] are represented as different colored blocks. N-terminal tails are drawn to scale with the relative locations of each motif identified. The height of the motif block is proportional to-log(p-value). (B) Motif blocks 4 and 6 in Logos format. All instances where the motifs were identified are included below for comparison.

Since our genetic assays highlight a critical role of the N-terminal tail sequence in segregation and genome elimination, we were interested in identifying patterns in its sequence evolution. N-terminal tails of CENH3 proteins are hyper-variable both in their amino acid sequence and length, ranging from 23 amino acids (*Pisum sativum*) to 194 amino acids (*Brachypodium distachyon*). Thus, reconstructing the evolutionary history of N-terminal tails from alignments of distant CENH3 lineages is not possible. Instead, we decided to use an alignment free approach and used the motif search program MEME to identify short conserved blocks of sequence homology in the otherwise unstructured N-terminal tail. A similar approach investigating N-terminal tail evolution in Drosophila species identified three conserved blocks of homology shared by all CENH3 alleles in that clade [26]. Our analysis of N-terminal tails includes variation from a significantly broader evolutionary timescale, with CENH3 sequences ranging from green algae to flowering plants. We identified seven stretches of conserved protein sequences, which we have termed Blocks 1–7 (Fig. 5A, S3 Table). The over-representation of *Brassicaceae*-clade specific motifs (4 of 7 Blocks) is a reflection of our sampling bias, in which 22 of the 67 N-terminal tail sequences were from species within the *Brassicaceae*.

Several interesting patterns were immediately apparent: First, Block 1 and Block 2 were identified in nearly all plant CENH3s and in canonical Histone H3 (Fig. 5A). It appears that while the intervening sequence is highly variable in both length and content, the N- and C-terminus of N-terminal tails are evolving under strict constraint. These Blocks were not identified in *H*. *sapiens* CENH3. Second, in several instances where a species’ genome carries two copies of CENH3, there was differential retention of Blocks between the two copies, a situation analogous to sub-functionalization post gene duplication. For example, copy A of CENH3 in *Arabidopsis lyrata* is missing Block 6 but retained Block 3, while copy B is missing Block 3 but has retained Block 6. In *Hordeum vulgare*, the monocot-specific Block 7 is retained in copy A, but lost in copy B. Third, isolated Blocks were identified across long evolutionary distances (Fig. 5B). For example, *Brassicaceae*-specific Block 4 was absent in all other lineages but present in *V*. *vinifera*, a basal rosid. Similarly, Block 6 that is present in most, but not all, *Brassicaceae* species, was also identified in two distant rosid species, *Phaseolus vulgaris* and *Glycine max*. The most parsimonious explanation for this pattern is that sequences homologous to Block 4 and Block 6 were present in the N-terminal tail of the ancestral CENH3 and were selectively retained or lost in the different rosid species. These observations suggest a modular evolutionary pattern where the constraints on individual Blocks are independent of one another. An outcome of this might be that the N-terminal tails acquire lineage-specific configuration of Blocks, thereby generating combinatorial sequence diversity.

## Discussion

The results obtained in this study provide new and dramatically different information about CENH3 function and evolution from that previously available [21]. We observed wide complementation of a *CENH3* loss-of-function mutation, while previous studies failed to obtain complementation except in the case of CENH3 from a very close relative. The difference lies quite simply in the use of untagged versus GFP-tagged CENH3 proteins in functional complementation assays. Furthermore, a recent study of CENH3^CSE4^ dynamics in yeast found that fusion of the GFP-tag to the CENH3^CSE4^ protein altered its function [27]. Taken together, it is apparent that presence of the GFP-tag significantly interferes with centromere function and protein modified with this fusion has limited use as a proxy for wild-type CENH3 activity.

The role of CENH3 in centromere determination is thought to have originated in an early eukaryotic ancestor [28]. Functional homologs of CENH3 have been identified in plants, animals, fungi and protists [29,30]. This essential gene exists as a single copy in nearly all species. Given the absence of gene duplicates and opportunities for sub-functionalization, this diversity in CENH3 protein sequences is puzzling and begs the question: how conserved are the functional requirements for making a centromere? This question has been asked in at least four different model organisms using primarily two assays: localization of evolutionarily distant CENH3s to the endogenous centromere and functional complementation of the endogenous CENH3 with evolutionary variants [18,21,31–33].

Two contrasting patterns of CENH3 functional conservation are apparent from the literature and this study. The first pattern is one of shared constraint over long evolutionary distances and the second is that of extreme lineage-specificity. In mammalian cells, GFP-tagged CENH3s from *C*. *elegans* and *S*. *cerevisiae* localized to centromeres. In addition, *S*. *cerevisiae* CENH3 rescued mammalian cells from mitotic arrest induced by depletion of the endogenous CENH3 [18]. In *Arabidopsis*, centromeric localization of complementing CENH3 does not extend as far as yeast [21] but CENH3 from *Z*. *mays*, a distant monocot species, can functionally substitute for the endogenous CENH3 (Fig. 4B and S6). In contrast, in *D*. *melanogaster*, GFP-tagged CENH3 from a species within the same genus failed to localize to centromeres [31]. In budding yeast, functional complementation of CENH3 is limited to the closely related hemiascomycetes [33]. Hemiascomycetes are unique in having ‘point centromeres’ that are genetically defined by a 125-bp sequence. Point centromeres are a derived evolutionary characteristic [28,34] and a plausible argument is that this specialized centromeric structure places severe lineage-specific constraints on CENH3 function, thereby restricting the limits of functional complementation. The results presented here argue that functional conservation despite sequence divergence is the norm, while stringent functional constraints might be symptomatic of a derived idiosyncratic centromere.

In this study we have asked not only whether a divergent CENH3 can functionally complement the endogenous *A*. *thaliana* allele, but also how well it complements those functions by providing a quantitative measure of the effect of CENH3 divergence on segregation fidelity. This measure has been possible because *A*. *thaliana*, like most plants, has a high tolerance for genomic dosage imbalance [35–37], thereby allowing recovery of the products of missegregation. Strikingly, complemented lines that had no fertility issues when fertilized by pollen of the same genotype, displayed large-scale segregation errors when crossed to wild type. Significant fractions of the recovered F1 progeny were either aneuploid or haploid (Table 1). In all cases the missegregated chromosomes originated from the parent expressing the divergent CENH3 (Fig. 3, S4 and S5). This clearly implies that centromeres built on the divergent CENH3, while able to complement essential functions, are deficient in comparison to the endogenous *A*. *thaliana* CENH3. What is the molecular basis of this functional deficiency? Answering this question constitutes an exciting next challenge since it will uncover species-specific adaptations to centromere function and shed light on what is driving the rapid evolution of this ancient biological structure.

Genome elimination as a barrier to interspecies hybridization has been observed in several taxa [38]. It had been previously shown that engineering modifications to CENH3, namely fusing an N-terminal GFP-tag and swapping the N-terminal domain with one from Histone H3.3 (GFP-tailswap), causes segregation errors and genome elimination. Our results now show that naturally occurring divergence in CENH3 has the same effect. The most parsimonious explanation is that the underlying mechanistic basis of genome elimination in these different systems is shared while differing quantitatively in its outcome. In contrast to the male-sterile GFP-tailswap construct, CENH3 evolutionary variants are perfectly fertile when selfed, imposing no obvious fitness cost per se (Fig. 1 and 2). This highlights the fact that unlike the artificial GFP-tailswap construct, the naturally occurring mutations in CENH3 have evolved under functional constraint and can fulfill the conserved, essential functions even in the context of a non-native centromere, at least under standard growth conditions. However, in crosses to gametes with wild-type centromeres, the difference in parental CENH3s produces inviable (aborted seeds) and sterile (aneuploid and haploid) F1 progeny. In addition to these fitness penalties, the cross creates genetic novelty including instances of chromosomal breakage and shuffling of the resulting segments (Fig. 3B-D).

Aneuploidy and elimination of the haploid inducer genome are likely a linked phenomenon. Interestingly, fragmented chromosomes have been observed in other systems where genome elimination follows from an interspecific hybridization event [39,40]. In the natural barley wide crosses and in wheat and pearl millet hybrids, micronuclei formation is observed during the process of genome elimination [39,41]. Chromosomes within micronuclei could be targeted for elimination or be rescued by the cell, resulting in potential aneuploid progeny. While most aneuploid karyotypes have a deleterious fitness effect, recent studies have shown that aneuploidy is able to confer adaptive phenotypes under various stress conditions [42,43]. In summary, our data strongly supports a role for CENH3 divergence in speciation, not only as a means for creating a postzygotic reproductive barrier but also as a driver of genetic novelty.

A major finding from our work is that it is divergence in the *L*. *oleraceum* N-terminal tail that is critical for the missegregation phenotype. Fusing *A*. *thaliana* N-terminal tail to a divergent HFD improved its function, while fusing a divergent N-terminal tail to the *A*. *thaliana* HFD corrupts its function. In fact this second chimera showed a more severe missegregation phenotype than the full-length divergent CENH3 (Table 1). This suggests that the two domains of CENH3 might be co-evolving with one another, thus in some cases a chimera between two non-adapted domains could create an allele that is worse than the sum of its individual parts. Nevertheless, our results show that, despite sequence divergence, the HFD of CENH3 from a distant species can be functionally interchanged. Domain-swap experiments have revealed that regions within the HFD are required for centromere localization [31,44]. A plausible hypothesis is that the structural and functional constraints on the HFD are essentially unchanging, while the N-terminal tail is evolving to accommodate lineage-specific differences in centromeric environment.

Our examination of N-terminal tail sequences across the plant kingdom suggests a pattern where blocks of sequence homology are being lost and gained in a lineage-specific manner (Fig. 5A). A tempting conjecture is that these blocks of homology represent functional modules, such as interactions with other centromere-associated proteins. If this was the case we could expect lineage-specific diversity in centromeric machinery, with the integration (or subtraction) of lineage-specific interactions into the ancestral centromere network. Consistent with this expectation, a recent study recently delineated the evolutionary trajectory of Umbrea, a neogene that has gained essential centromeric functions in specific Drosophila lineages [45]. While this is in no way conclusive, we propose that the idiosyncratic rewiring of centromeric chromatin constitutes a potential driving force for the evolution of the N-terminal tail of CENH3.

In summary, our results argue that while CENH3 from all species perform conserved functions, each CENH3 is adapted to its own unique cellular, most likely centromeric, environment. Why there should exist so many diverse solutions to the problem of packaging centromeric chromatin remains enigmatic. However, we demonstrate that this lineage-specific diversification of CENH3 has the potential to contribute to the genetic diversification and reproductive isolation of populations.

## Materials and Methods

### Plant materials and crossing procedure

Plants were transformed by the Agrobacterium floral dip method using standard protocols. Plants were grown under 16 hr of light/8 hr of dark at 20°C. For each cross, at least five flowers from an early inflorescence were emasculated and pollinated one day later with wild type pollen. F1 seeds were first sown in 0.5X MS plates containing 1% sucrose to maximize germination efficiency and then transplanted to soil.

### Cloning of CENH3 transgenes

The *L*. *oleracem* CENH3 coding region including introns was PCR amplified from genomic DNA with the addition of *Sal*I and *Xba*I sites at the ends. This PCR product was then cloned using standard restriction enzyme cloning into CP225, a cassette vector generated by Ravi *et al*. (2010) [21]. This vector is based on pCAMBIA1300 and carries the endogenous *A*. *thaliana* CENH3 promoter region i.e. 1489 bp upstream of the ATG, followed by a small linker region containing *Sal*I and *Xba*I sites and finally the CENH3 transcriptional terminator i.e. 585 bp downstream of the STOP codon.

All other constructs were cloned into a new Gateway-compatible cassette vector SM2 that was derived from the above CP225. To construct this vector, we used three-fragment multi-site gateway technology (Life technologies, cat# 12537–023) that allows simultaneous assembly of three DNA fragments in a defined order into a destination vector. The first and third fragments are the endogenous *A*. *thaliana* CENH3 promoter and terminator respectively, while the second fragment can be any CENH3 variant being tested. We PCR amplified the promoter and terminator sequences from CP225 flanked by the appropriate attB sites and recombined them via the BP reaction into pDONR 221 P1-P4 and pDONR 221 P3-P2 respectively, generating the following entry clones: pENTR L1-promoter-L4 and pENTR L3-terminator-L2. Next, we integrated these two along with pENTR R4-pLac-Spec-R3, the control entry clone for the second fragment, into the destination vector through a single LR reaction. The destination vector was a generous gift from the Pikaard Lab and was a modified pEARLEYGATE302 binary vector that has an additional ampicillin resistance gene for bacterial selection. We then did a reverse BP reaction with this intermediate expression plasmid and pDONR 221 P4r-P3r to replace the placeholder in the second fragment with the Gateway negative selection cassette [Cm^R^-*ccd*B] generating the final cassette vector, SM2 = CENH3 promoter-attL4-Cm^R^-*ccd*B-attL3-terminator in pEARLEYGATE302.

The *B*. *rapa* CENH3 genomic sequence was PCR amplified from the GFP-tagged *B*. *rapa* CENH3 plasmid generated in Ravi et al (2010) [21]. A chimeric transgene combining the *A*. *thaliana* N-terminal tail domain with *L*. *oleraceum* HFD was constructed by overlapping PCR. The N-terminal domain included genomic sequence coding for CENH3 starting from the “ATG” up to but not including the “PGTVAL” motif and the HFD extended from the “PGTVAL” motif to the STOP codon. The reciprocal construct with *L*. *oleraceum* N-terminal tail domain and *A*. *thaliana* HFD was similarly constructed. Transgenic variants outside the Brassicaceae were generated using CENH3 cDNA. *Z*. *mays* CENH3 was PCR amplified from plasmid generated in Ravi et al (2010) [21]. CENH3 cDNA from *V*. *vinifera* was synthesized by GenScript USA Inc. Piscataway, NJ based on the Genbank sequence, 225454488.

### DNA extraction and genotyping

Genomic DNA preparation and PCR genotyping were performed using standard methods. *cenh3–1* was genotyped with dCAPS primers. To genotype the *cenh3–1* mutation in lines with the construct *A*. *thaliana* N-terminal tail domain fused to *L*. *oleraceum* HFD, we first performed a PCR reaction with one primer outside the CENH3 promoter genomic DNA fragment present in the transgene. This PCR product was then used as the template in the dCAPS genotyping reaction. For each construct transgene-specific PCR primers were designed and used to confirm the genotype of each transgenic line. Primer sequences are available on request.

### Vegetative growth and fertility assays

Representative images of rosettes were taken 25 to 30 days after germination. The percentage of normal seeds was determined by visual inspection using a dissecting microscope. On average, seeds from five individual siliques were pooled and counted for one individual from each T1 family identified as CENH3 transgene +/+ *cenh3* -/-. Alexander staining of anthers was done according to published protocols [46].

### Meiotic chromosome spreads

DAPI stained male meiotic chromosome spreads were prepared as described in Ross et al. [47], and imaged using an Olympus BX61 epifluorescence microscope and Digital Scientific SmartCapture 3 software

### Characterization of haploids and aneuploids

Flow cytometric determination of genome content was performed on floral buds using published protocols [48]. 0.1g leaf tissue from aneuploid plants were collected and purified using DNA Phytopure Kit (GE). Genomic DNA libraries were prepared using the standard NEB Next DNA Library Prep with NEXTFlex-96 Adapters from BIOO Scientific, pooled and sequenced on Illumina HiSeq 2000 for 50bp single reads. The resulting reads were mapped to TAIR10 using BWA followed by chromosome dosage analysis using the protocol described in Henry et al (2010) [23]. All the individuals that were sequenced and analyzed are identified with a unique FRAG identifier and are described in S2 Table.

### Phylogenetic analysis

Reference IDs for all sequences used in this study are available in S1 Table. Multiple alignments of protein sequences encoding the histone fold domain of CENH3s was generated using MUSCLE and refined manually [49]. Evolutionary analyses were conducted in MEGA6 [50]. Phylogenetic history was inferred using the Maximum Likelihood method. The analysis involved 71 protein sequences. All positions containing gaps and missing data were eliminated. There were a total of 85 positions in the final dataset.

### Motif identification

MEME [51] with default parameters was used to identify statistically significant blocks of sequence homology in N-terminal tails extracted from 67 plant CENH3 sequences available from public databases.

## Supporting Information

S1 Fig (A) Meiotic prophase I in *L*. *oleraceum* CENH3 complemented lines. Meiotic prophase I is divided into 5 cytologically distinct sub-stages. Chromosomes are associated with a proteinaceous axis during leptotene. Axes of homologous chromosomes juxtapose together during zygotene, as a protein structure called the synaptonemal complex polymerizes between them. Synapsis is complete at pachytene, where homologues are fully paired. Homologues begin to separate during diplotene, but remain associated by chiasmata, marking the points of genetic crossover generated by homologous recombination. Chromosomes are condensed further at diakinesis, where chiasmata are more readily visible. In LoCENH3 (-/-), prophase I is cytologically indistinguishable from wild type, indicating that the complementation does not affect meiotic recombination. Scale bar = 10μm. (B) Chromosome fragmentation was observed in a single anaphase II pollen mother cell. Chromosome fragments are indicated by arrows. Scale bar = 10μm
(PDF)

S2 Fig Confirmation of haploid genome content in phenotypic haploids. (A) Representative haploid plant. Note absence of silique elongation and trichomeless leaves associated with recessive *gl1–1* glabrous mutation. (B) Comparison of nuclear DNA content of flower buds from 4 wild-type diploids and 11 phenotypic haploids as determined by flow cytometry.
(TIF)

S3 Fig Absence of phenotypic abnormalities in selfed populations of CENH3 complemented lines. Selfed progeny of CENH3 complemented lines are phenotypically similar to WT Col-0 plants, in contrast to the selfed triploid population that exhibits phenotypic diversity due to expected aneuploidy. LoCENH3 is *L*. *oleraceum* CENH3 and BrCENH3 is *B*. *rapa* CENH3. The genotype of the endogenous *CENH3* locus is indicated in parentheses.
(TIF)

S4 Fig Dosage plots and SNP analysis using whole genome sequencing of diploids and aneuploids from *L*. *oleraceum* CENH3 genome elimination crosses. (A) Dosage plots with 100kb bins across all five chromosomes. (B) Percent Col-0 SNPs across a 1Mb region across all five chromosomes.
(TIF)

S5 Fig Dosage plots and SNP analysis using whole genome sequencing of diploids and aneuploids from *B*. *rapa* CENH3 genome elimination crosses. (A) Dosage plots with 100kb bins across all five chromosomes. (B) Percent Col-0 SNPs across a 1Mb region across all five chromosomes.
(TIF)

S6 Fig Phenotype of CENH3 complemented lines. (A) Shown here are plants of the same age. (B) Confirmation of genotype by PCR. The genotype of the endogenous *CENH3* locus is indicated in parentheses. LoCENH3 is *L*. *oleraceum* CENH3, BrCENH3 is *B*. *rapa* CENH3, VvCENH3 is *V*. *vinifera* CENH3 and ZmCENH3 is *Z*. *mays* CENH3. AtNTT-LoHFD is a chimeric CENH3 where the *A*. *thaliana* N-terminal tail is fused to the *L*. *oleraceum* HFD and LoNTT-AtHFD is the reciprocal construct.
(TIF)

S7 Fig Alignment of CENH3 Histone Fold Domain protein sequences. Positions identical to *A*. *thaliana* are represented as (.) and positions different from *A*. *thaliana* are indicated by the corresponding amino-acid substitution.
(TIFF)

S1 Table List of all gene sequences and their database IDs used in this study. (DOCX)

S2 Table Characteristics of all aneuploids analyzed by whole genome sequencing in this study. (XLS)

S3 Table List of all sequences identified by MEME as motifs. (XLSX)
