# Supplementary figures and images for: Naturally Occurring Differences in CENH3 Affect Chromosome Segregation in Zygotic Mitosis of Hybrids
Source: PLoS Genet. 2015 Jan 26;11(1):e1004970. doi: 10.1371/journal.pgen.1004970 (PMC4314295; doi:10.1371/journal.pgen.1004970)

**Figure S1A**

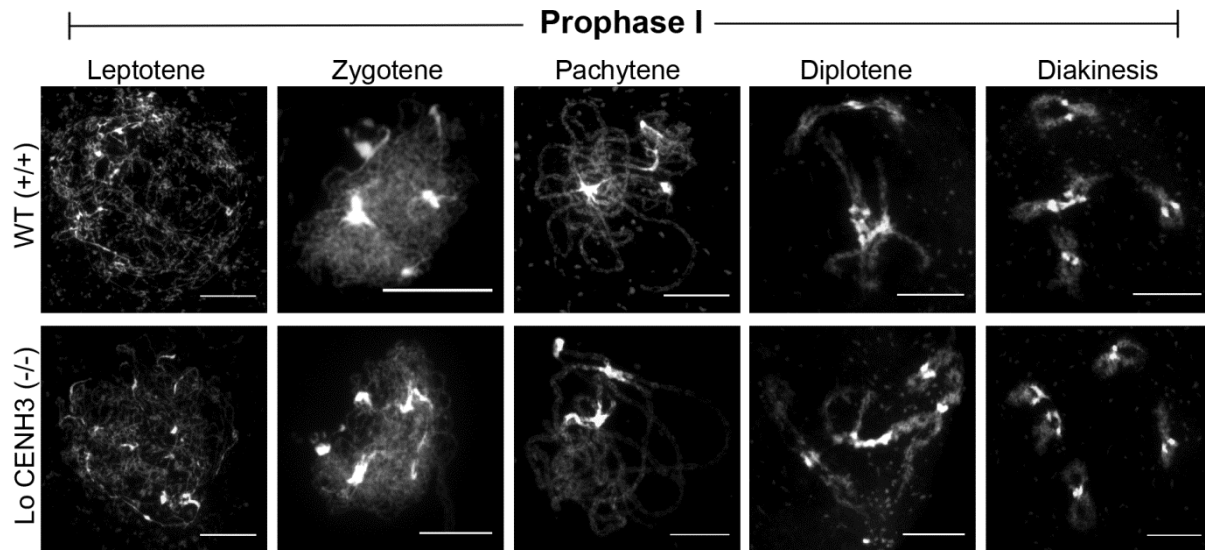

**Figure S1B**

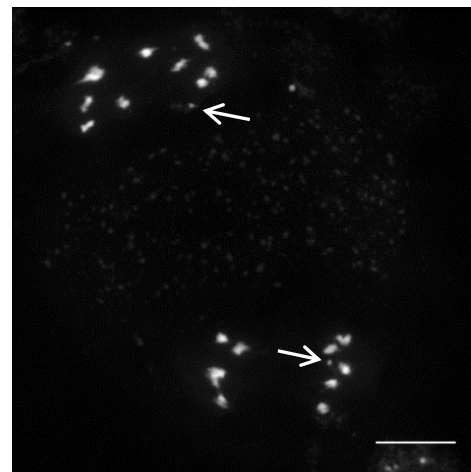

Supplement: S1 Fig — Meiotic prophase I is divided into 5 cytologically distinct sub-stages. Chromosomes are associated with a proteinaceous axis during leptotene. Axes of homologous chromosomes juxtapose together during zygotene, as a protein structure called the synaptonemal complex polymerizes between them. Synapsis is complete at pachytene, where homologues are fully paired. Homologues begin to separate during diplotene, but remain associated by chiasmata, marking the points of genetic crossover generated by homologous recombination. Chromosomes are condensed further at diakinesis, where chiasmata are more readily visible. In LoCENH3 (-/-), prophase I is cytologically indistinguishable from wild type, indicating that the complementation does not affect meiotic recombination. Scale bar = 10μm. (B) Chromosome fragmentation was observed in a single anaphase II pollen mother cell. Chromosome fragments are indicated by arrows. Scale bar = 10μm (PDF) [file pgen.1004970.s001.pdf]

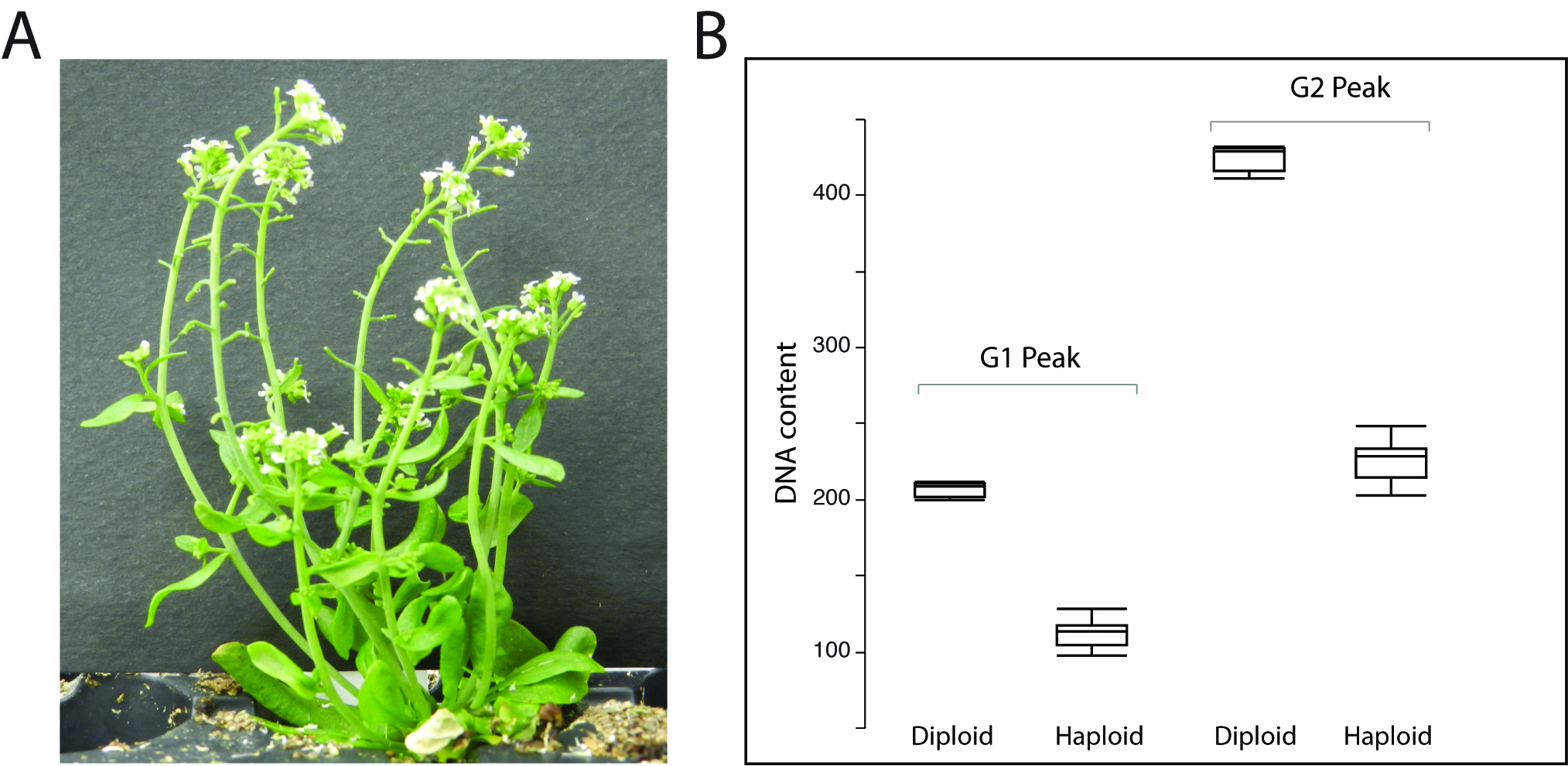

Supplement: S2 Fig — (A) Representative haploid plant. Note absence of silique elongation and trichomeless leaves associated with recessive gl1–1 glabrous mutation. (B) Comparison of nuclear DNA content of flower buds from 4 wild-type diploids and 11 phenotypic haploids as determined by flow cytometry. (TIF) [file pgen.1004970.s002.tif]

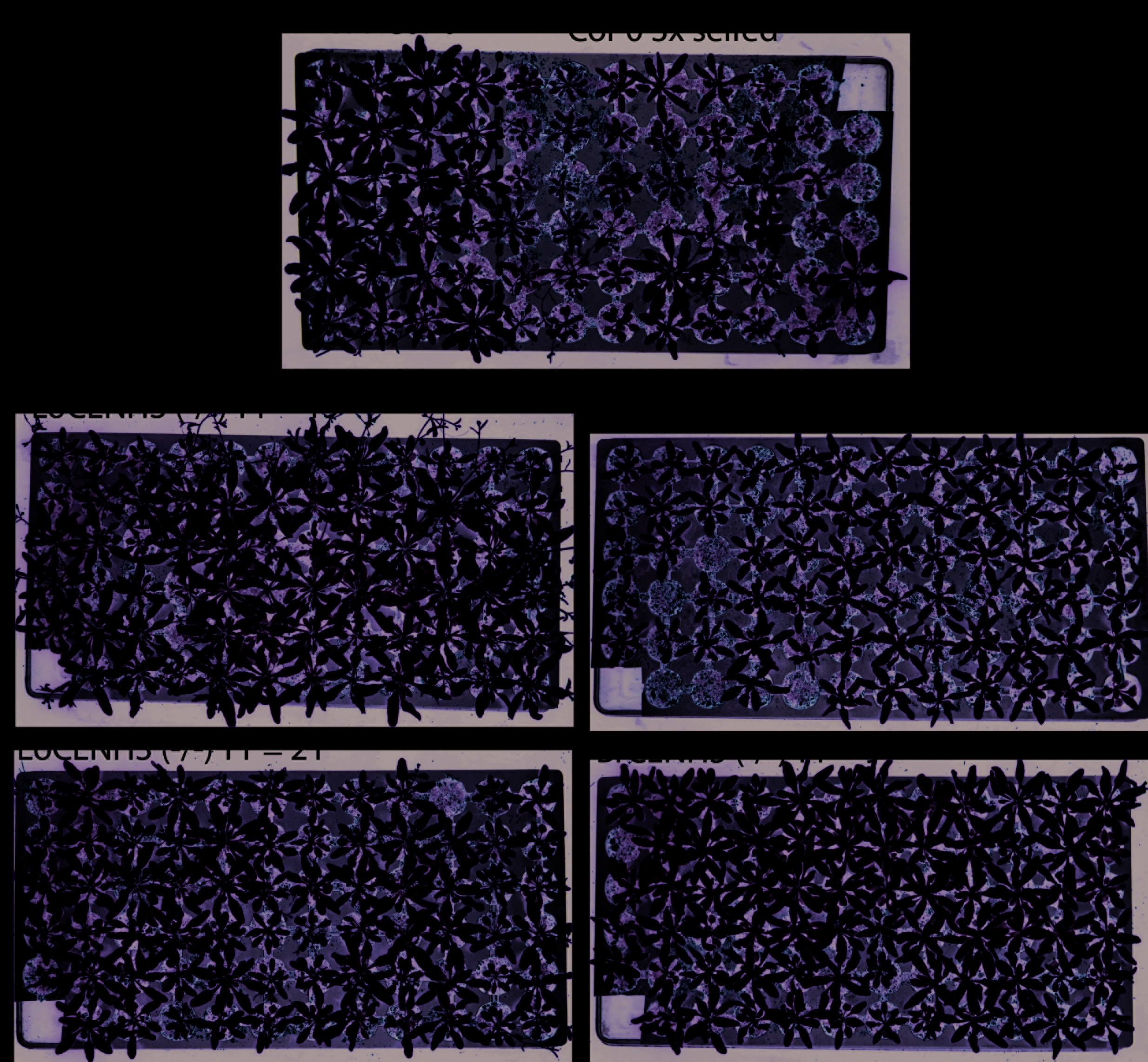

Supplement: S3 Fig — Selfed progeny of CENH3 complemented lines are phenotypically similar to WT Col-0 plants, in contrast to the selfed triploid population that exhibits phenotypic diversity due to expected aneuploidy. LoCENH3 is L. oleraceum CENH3 and BrCENH3 is B. rapa CENH3. The genotype of the endogenous CENH3 locus is indicated in parentheses. (TIF) [file pgen.1004970.s003.tif]

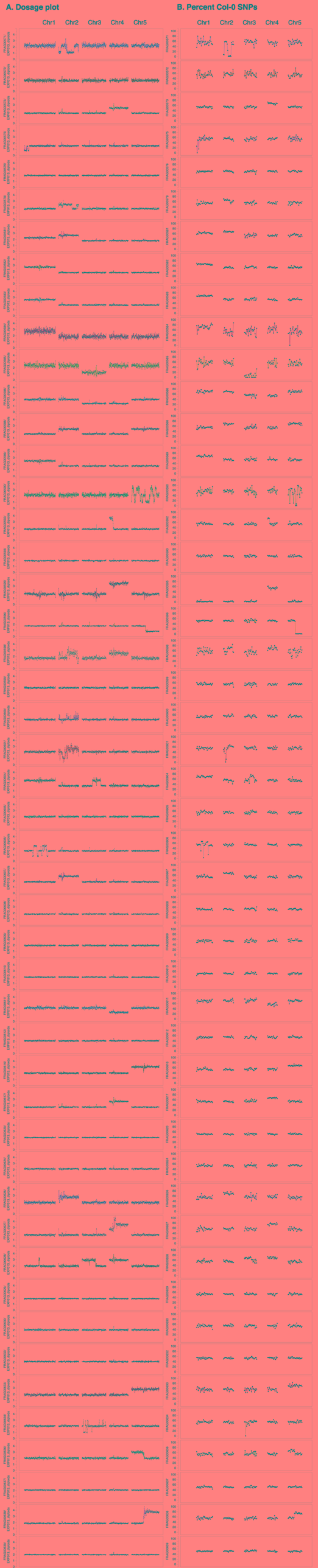

Supplement: S4 Fig — (A) Dosage plots with 100kb bins across all five chromosomes. (B) Percent Col-0 SNPs across a 1Mb region across all five chromosomes. (TIF) [file pgen.1004970.s004.tif]

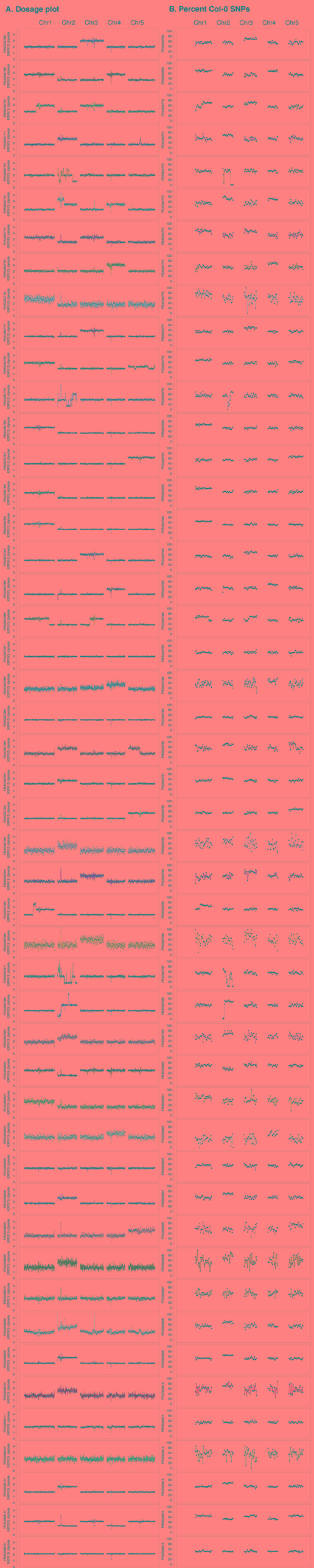

Supplement: S5 Fig — (A) Dosage plots with 100kb bins across all five chromosomes. (B) Percent Col-0 SNPs across a 1Mb region across all five chromosomes. (TIF) [file pgen.1004970.s005.tif]

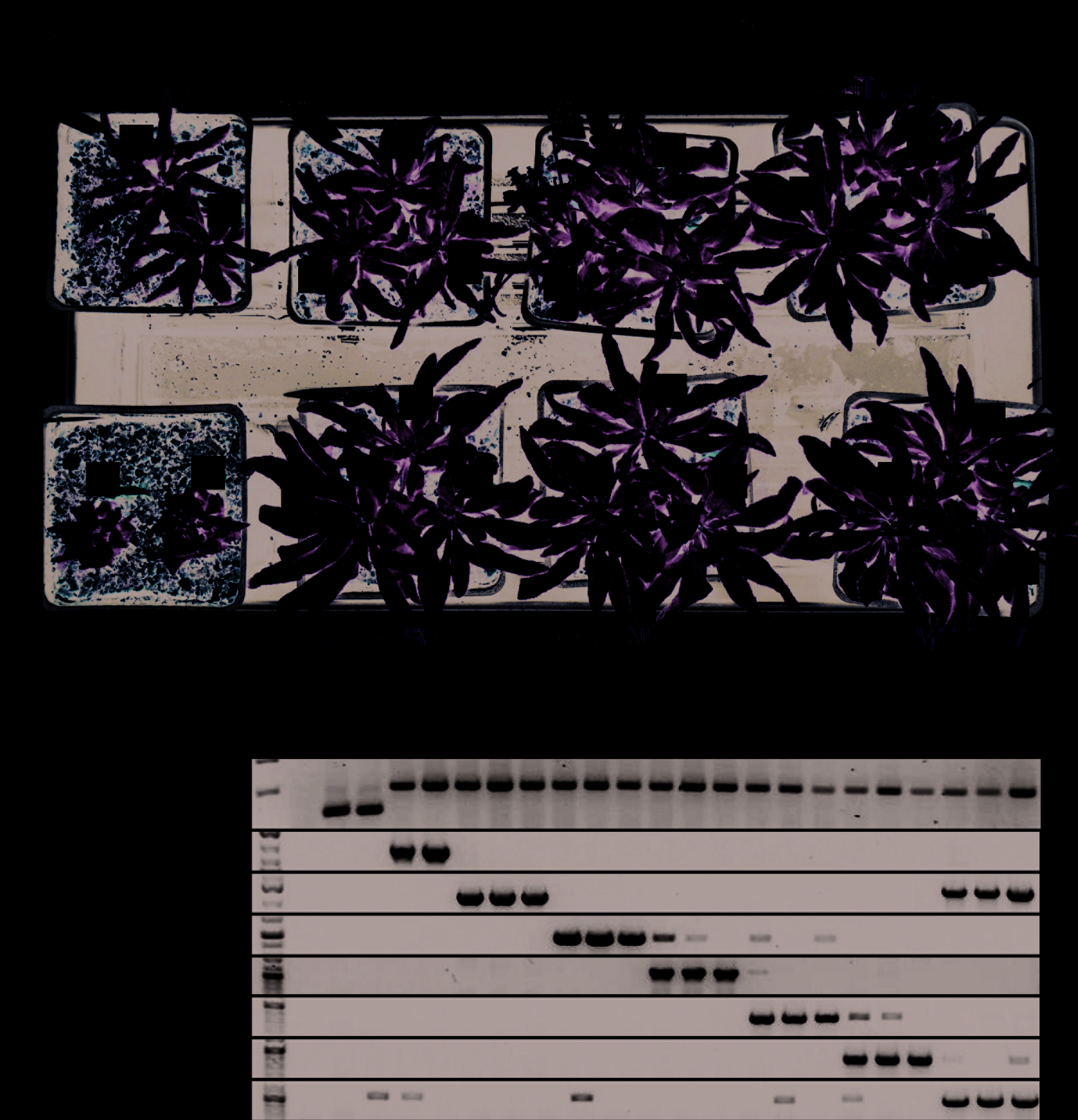

Supplement: S6 Fig — (A) Shown here are plants of the same age. (B) Confirmation of genotype by PCR. The genotype of the endogenous CENH3 locus is indicated in parentheses. LoCENH3 is L. oleraceum CENH3, BrCENH3 is B. rapa CENH3, VvCENH3 is V. vinifera CENH3 and ZmCENH3 is Z. mays CENH3. AtNTT-LoHFD is a chimeric CENH3 where the A. thaliana N-terminal tail is fused to the L. oleraceum HFD and LoNTT-AtHFD is the reciprocal construct. (TIF) [file pgen.1004970.s006.tif]

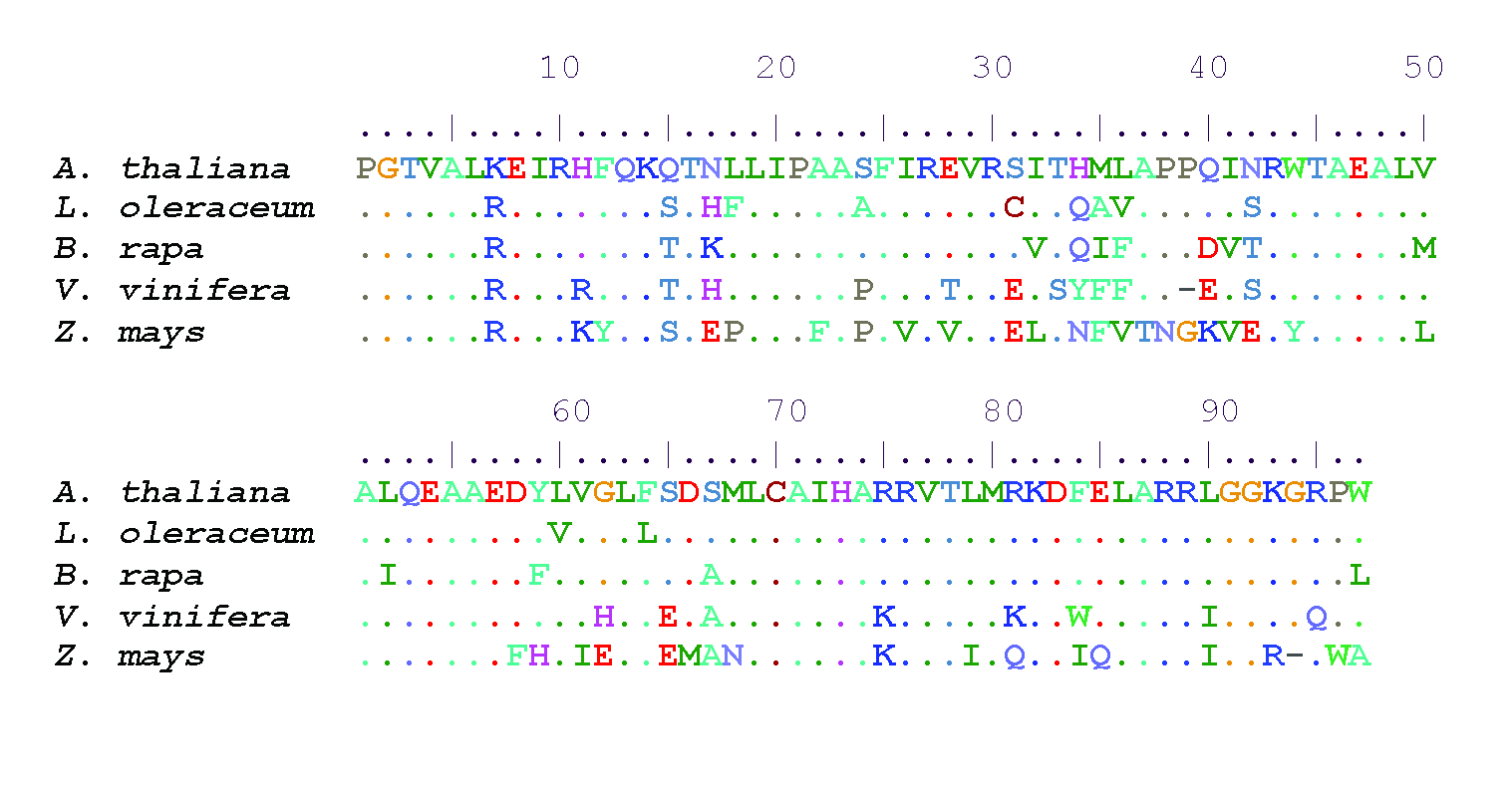

Supplement: S7 Fig — Positions identical to A. thaliana are represented as (.) and positions different from A. thaliana are indicated by the corresponding amino-acid substitution. (TIFF) [file pgen.1004970.s007.tiff]
