# Supplementary material for: Naturally Occurring Differences in CENH3 Affect Chromosome Segregation in Zygotic Mitosis of Hybrids
Source: PLoS Genet. 2015 Jan 26;11(1):e1004970. doi: 10.1371/journal.pgen.1004970 (PMC4314295; doi:10.1371/journal.pgen.1004970)
Supplement: S1 Table — (DOCX) [file pgen.1004970.s008.docx]

**Table S1. A list of all gene sequences and their database IDs used in this study.**

|  | **Source of Sequence** | **Sequence Identifier** | **Database** |
| --- | --- | --- | --- |
| 1 | Arabidopsis arenosa | AF465801 | Genbank |
| 2 | Arabidopsis halleri A | AB081501 | Genbank |
| 3 | Arabidopsis halleri B | AB081503 | Genbank |
| 4 | Arabidopsis lyrata A | DQ450587 | Genbank |
| 5 | Arabidopsis lyrata B | DQ450557 | Genbank |
| 6 | Arabidopsis thaliana | AB081500 | Genbank |
| 7 | Arabis hirsuta | AB299169 | Genbank |
| 8 | Brassica nigra | GU166738 | Genbank |
| 9 | Brassica oleracea | GU166739 | Genbank |
| 10 | Brassica rapa | GU166737 | Genbank |
| 11 | Capsella bursa-pastoris | AB299175 | Genbank |
| 12 | Capsella rubella | XM_006304182 | Genbank |
| 13 | Cardamine flexuosa | AB299171 | Genbank |
| 14 | Crucihimalaya himalaica | AY612790 | Genbank |
| 15 | Crucihimalaya wallichii | AB299177 | Genbank |
| 16 | Eruca sativa | AB299180 | Genbank |
| 17 | Lepidium oleraceum |  | This study, being submitted to Genbank |
| 18 | Lepidium virginicum | AB299181 | Genbank |
| 19 | Olimarabidopsis pumila | AB299167 | Genbank |
| 20 | Raphanus sativus | AB299183 | Genbank |
| 21 | Thellungiella halophila | Thhalv10008889m | Phytozome |
| 22 | Turritis glabra | AB081505 | Genbank |
| 23 | Astragalus sinicus | AB649144 | Genbank |
| 24 | Carica papaya | EX259948 | Genbank |
| 25 | Citrus sinensis | XM_006491184 | Genbank |
| 26 | Cucumis sativus | XM_004139613 | Genbank |
| 27 | Eucalyptus grandis | Eucgr.D00189.1 | Phytozome |
| 28 | Fragaria vesca | XM_004306639 | Genbank |
| 29 | Glycine max | XM_003528751 | Genbank |
| 30 | Gossypium raimondii | Gorai.001G155600.1 | Phytozome |
| 31 | Linum usitatissimum | Lus10008119 | Phytozome |
| 32 | Lotus japonicus | BT137822 | Genbank |
| 33 | Malus domestica | MDC013092 | Phytozome |
| 34 | Manihot esculenta | FF379687 | Genbank |
| 35 | Medicago truncatula | XM_003637685 | Genbank |
| 36 | Phaseolus vulgaris | KC491791 | Genbank |
| 37 | Pisum Sativum A | JF739989 | Genbank |
| 38 | Pisum Sativum B | JF739990 | Genbank |
| 39 | Populus trichocarpa | XM_002320818 | Genbank |
| 40 | Theobroma cacao | XM_007051531 | Genbank |
| 41 | Vitis vinifera | XM_002281037 | Genbank |
| 42 | Mimulus guttatus | GR117778 | Genbank |
| 43 | Nicotiana sylvestris | AB467328 | Genbank |
| 44 | Nicotiana tomentosiformis | AB467329 | Genbank |
| 45 | Solanum lycopersicum | BG127218 | Genbank |
| 46 | Solanum tuberosum | XM_006339625 | Genbank |
| 47 | Allium cepa | AB600275 | Genbank |
| 48 | Allium fistulosum | AB571555 | Genbank |
| 49 | Allium sativum | AB571556 | Genbank |
| 50 | Allium tuberosum | AB571557 | Genbank |
| 51 | Brachypodium distachyon | XM_003566059 | Genbank |
| 52 | Hordeum bulbosum A | GU245882 | Genbank |
| 53 | Hordeum bulbosum B | JF419330 | Genbank |
| 54 | Hordeum vulgare A | JF419328 | Genbank |
| 55 | Hordeum vulgare B | JF419329 | Genbank |
| 56 | Luzula nivea A | AB201356 | Genbank |
| 57 | Luzula nivea B | HM988988 | Genbank |
| 58 | Oryza sativa | AY438639 | Genbank |
| 59 | Panicum virgatum | FL730019 | Genbank |
| 60 | Saccharum officinarum | CA127217 | Genbank |
| 61 | Setaria italica | XM_004961625 | Genbank |
| 62 | Sorghum bicolor | XM_002441245 | Genbank |
| 63 | Zea mays | NM_001112050 | Genbank |
| 64 | Physcomitrella patens | XM_001785914 | Genbank |
| 65 | Chlamydomonas reinhardtii | XM_001697817 | Genbank |
| 66 | Coccomyxa subellipsoidea | XM_005647320 | Genbank |
| 67 | Micromonas pusilla | XM_003056465 | Genbank |
| 68 | Arabidopsis thaliana H3.1 | NM_125934 | Genbank |
| 69 | Homo sapiens H3.1 | X57128 | Genbank |
| 70 | Homo sapiens CENH3 | NM_001809 | Genbank |
